# Projected AR Serious Game “Painting Discovery” for Shoulder Rehabilitation: Assessment With Technicians, Physiotherapists, and Patients

**DOI:** 10.1109/JTEHM.2025.3557250

**Published:** 2025-04-02

**Authors:** Giuseppe Turini, Marina Carbone, Sara Condino, Donato Gallone, Vincenzo Ferrari, Marco Gesi, Michelangelo Scaglione, Paolo Parchi, Rosanna Maria Viglialoro

**Affiliations:** Computer Science DepartmentKettering University 3364 Flint MI 48504 USA; EndoCAS Interdeparmental Center for computer Assisted SurgeryUniversity of Pisa 9310 56125 Pisa Italy; Department of Information EngineeringUniversity of Pisa 9310 56122 Pisa Italy; Department of Orthopaedics and Trauma SurgeryUniversity of Pisa 9310 Pisa 56100 Italy; Department of Translational Research and of New Surgical and Medical TechnologiesUniversity of Pisa 9310 56126 Pisa Italy

**Keywords:** Hand-finger tracking, projected augmented reality, rehabilitation, serious game, shoulder disorders. Clinical Impact: “Painting Discovery” could significantly impact clinical practice by improving patient adherence through increased engagement and motivation. This may reduce the need for supervised sessions, making rehabilitation more accessible and enhancing overall effectiveness

## Abstract

Objective: Motivation and adherence are crucial for effective rehabilitation, yet engagement remains a challenge in upper limb physiotherapy. Serious Games (SGs) have emerged as a promising tool to enhance patient motivation. This study evaluates Painting Discovery, a projected augmented reality (AR) SG for shoulder rehabilitation, assessing engagement, ergonomics, and its potential to differentiate motor performance between healthy and those with rheumatoid arthritis, bursitis, subacromial impingement, rotator cuff tear, or calcific tendinopathy. Additionally, it examines improvements in pathological subjects following physiotherapy. Method: Sixteen healthy and seven pathological subjects participated. Engagement, ergonomics, and satisfaction were assessed using Likert-scale questionnaires. Motor performance was evaluated through completion time, speed, acceleration, and normalized jerk. Four pathological subjects underwent pre- and post-physiotherapy assessments over six weeks. Results: SG was highly engaging and ergonomic, with no significant differences based on prior video game or AR experience. The pathological group had longer completion times ( $56.49~\pm ~37.85$s vs. $39.02~\pm ~24.21$s, p < 0.001), lower acceleration ( $1.11~\pm ~0.92$ m/s2 vs. $0.79~\pm ~0.56$ m/s2, p < 0.001), and higher jerk ( $6.68\times 107~\pm ~1.37\times 108$ m/s3 vs. $9.22\times 106~\pm ~2.51\times 107$ m/s3, p = 0.025) then healthy subjects. After physiotherapy, completion time and normalized jerk indicated enhanced efficiency and control. Conclusions: Painting Discovery shows strong potential as an engaging, accessible rehabilitation tool. While effective in differentiating motor impairments, its small sample size and horizontal-plane movement focus limit broader conclusions. Future studies should expand participation, incorporate vertical-plane movements, and refine performance metrics for clinical validation.

## I. Introduction

Musculoskeletal disorders are a leading cause of joint pain and physical disability worldwide, with chronic shoulder pain causing a significant socioeconomic impact [1], [2]. Rehabilitation, including physical therapy, exercise, psychotherapy, sports activity, and alternative medicine, is a critical part of treatment alongside medication and surgery [3]. Physiotherapy focuses on correcting muscle weaknesses, dysfunctions, stiffness, and postural abnormalities [4], while post-operative rehabilitation prevents stiffness and strengthens muscles.

Patient motivation and adherence to treatment are crucial for effective rehabilitation [5], yet only 50% of patients with chronic diseases adhere to their treatment plans [6]. Upper limb physiotherapy is generally less effective than lower limb physiotherapy due to the priority given to restoring walking ability and the complications of shoulder rehabilitation, such as pain and subluxations [7]. The high incidence of shoulder conditions and low adherence to long-term therapy place a significant burden on the healthcare system [2].

Gamification, the application of game-like elements, is increasingly recognized as a strategy to enhance motivation and adherence in long-term rehabilitation [8]. In recent years, researchers have developed SGs specifically for motor rehabilitation of the upper limb [9]. In rehabilitation, studies have shown that SGs, particularly exergames (i.e., games requiring physical exercise to play) can effectively address the low engagement seen in conventional physiotherapy and offer a cost-effective solution [10], [11], [12]. For example, Dahl-Popolizio et al. [13] developed a Kinect-based SG that engages patients in task-specific reaching and grasping exercises to enhance motor control and improve muscle strength. Similarly, in [14], the authors demonstrated that a virtual reality exergaming platform can yield short-term improvements in upper limb function for patients with conditions such as subacromial impingement syndrome. In addition, González-González et al. [10]introduced a SG platform that incorporates gestural interaction and a recommender system to deliver personalized gamified exercises, indicating potential benefits for motor control in upper limb rehabilitation. Furthermore, Pirovano et al. [11], proposed a comprehensive methodology for designing effective and safe therapeutic exergames—a framework that has been applied to rehabilitation protocols to facilitate motor recovery in the upper limb.

Interaction modalities in SGs for upper limb rehabilitation vary widely, from traditional mouse/keyboard setups to advanced VR and AR interfaces, including VR/AR controllers, gesture-based interactions, and haptic interfaces [15]. However, these innovative interfaces often require technology (sensors, controllers, etc.) that can be challenging for patients with varying levels of motor disability to use or wear. Additionally, the high cost and need for specific configuration skills limit their use of specialized rehabilitation centers.

Video game technology has recently emerged as a significant player in rehabilitation, particularly with the advent of full-body tracking devices, and hand and finger tracking systems like the Leap Motion Controller (LMC). This technology is rapidly gaining attention and proving its potential in significantly enhancing rehabilitation processes, offering a hopeful future for healthcare.

These health-focused games have prompted the development of quality criteria that combine both ‘serious’ and ‘game’ parameters to ensure their effectiveness and engagement [16].

**Game Parameters:**

• **Fun:** Involvement, continuity (dynamic adaptation to skills), establishing emotional, control sense, social interactions (competitive or collaborative settings), and immersive experience.
• **Presentation:** Eye-catching graphics and proper audio.

**Serious Parameters:**

• **Characterizing Objective:** Focus on the objective (the user should remain concentrated), clear goals, indispensability of the characterizing objective.
• **Methods:** Absence of game errors, appropriate progress feedback, positive reinforcement.
• **Quality:** Proof of effectiveness and sustainability in achieving and maintaining the result is crucial. Rewards and rankings play a significant role in motivating players and enhancing their gaming experience.

This study marks the initial stride towards clinically validating our projected AR serious game for shoulder rehabilitation, a potential game-changer in the field.

In the previous works, we focused on developing the software and hardware of the system and identifying the most suitable technologies [17], [18], [19].

Unlike other SGs, our system introduces key innovations for shoulder rehabilitation by combining real-time auditory and visual feedback with customizable gameplay to enhance engagement and therapeutic effectiveness. Instructions, scores, and completion times are displayed during sessions, while background music and error-specific audio cues enhance engagement and motor learning. Unlike TANGO:H which lacks real-time feedback [10], our system provides continuous performance guidance. Customization options allow therapists to adjust trajectory paths, task sequences, and desk pad friction to meet individual patient needs—an advantage over Wii Exergaming [11], which uses standardized tasks. The system’s portability and low cost, enabled by the LMC, support home-based training with minimal supervision. This lightweight, sensor-free design improves accessibility for users with motor impairments, unlike Wii Fit, which requires handheld controllers.

Overall, our game combines adaptability, affordability, and engaging feedback mechanisms, providing an accessible and effective tool for shoulder rehabilitation across diverse patient populations.

In [15], we presented the first prototype of our projected AR SG and tested its functionality with 16 healthy subjects, including rehabilitation and technical experts (with positive evaluations of both ergonomics and user involvement).

The main objectives of the present study are:

1. Confirming the good involvement and ergonomics in healthy subjects and subjects with shoulder pathology, and comparing patients’ acceptance of this system in respect to traditional physiotherapy.
2. Assessing whether the performance parameters can distinguish a pathological limb from a healthy one and verify a possible improvement of the pathological limb after traditional physiotherapy.
3. Identifying and then including the ideal population in a future rehabilitation protocol based on the “Painting Discovery” SG and evaluating the importance of the experience with video games and AR and the role of the degree of disability.

## II. Materials and Methods

We conducted a prospective controlled observational study in collaboration with the EndoCAS Research Center and the “Sport and Anatomy” Center for Rehabilitation Medicine and Sport Medicine of the University of Pisa. Approved by the Bioethics Committee of the University of Pisa (Opinion No. 38/2022), the study posed no risks to participants, and we processed all data according to privacy regulations.

### A. Features of the Projected AR Serious Game for Shoulder Rehabilitation: “Painting Discovery”

**FIGURE 1.**
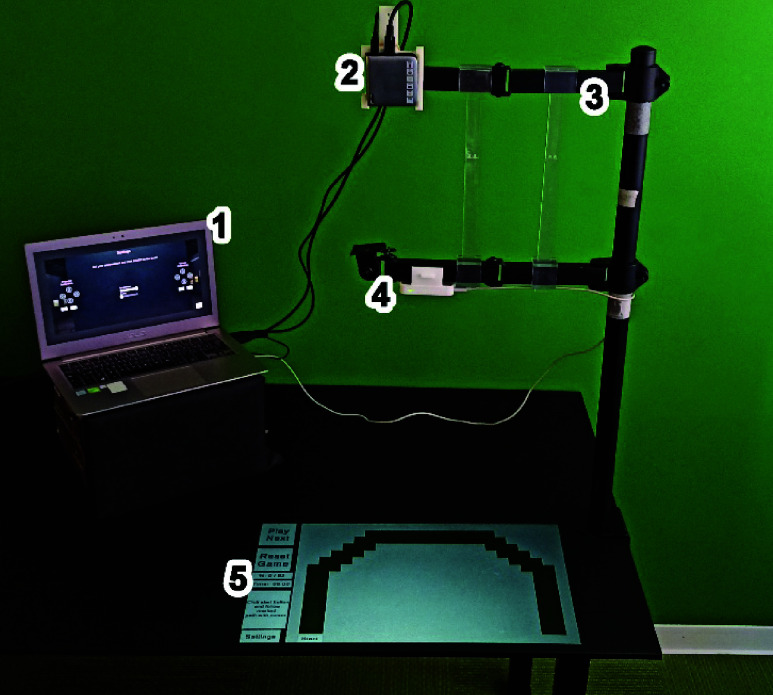
Hardware components of the SG “Painting Discovery”: 1) Laptop,2).PicoPix projector,3) desk-anchored metallic structure with two adjustable arms,4) LMC, and 5)black rubber desk mat.

The hardware components of our SG include an LMC [20], a commercially available hand-tracking device equipped with two infrared (IR) depth-sensing cameras and three IR LEDs, enabling real-time tracking of hand movements. Additionally, a portable PicoPix projector [21] (Philips PPX4010) is used to project the SG in AR including the virtual cursor that follows the patient’s hand. The system comprises a desk-anchored metallic structure with two adjustable arms to support and orientate both the LMC and the projector, an ad-hoc 3D-printed supports to hold the projector and LMC in place on the metallic arms, a projection surface (black rubber desk mat, size $60\times 40$cm), and a laptop PC (ASUS UX303UB) running the SG (Figure 1). The software was developed in C# using the Unity 3D game engine (version 2019.4.18f1) for the Microsoft Windows 10 platform and integrating the Leap Motion Orion Beta (version 4) by using its plugin for Unity.

**FIGURE 2.**
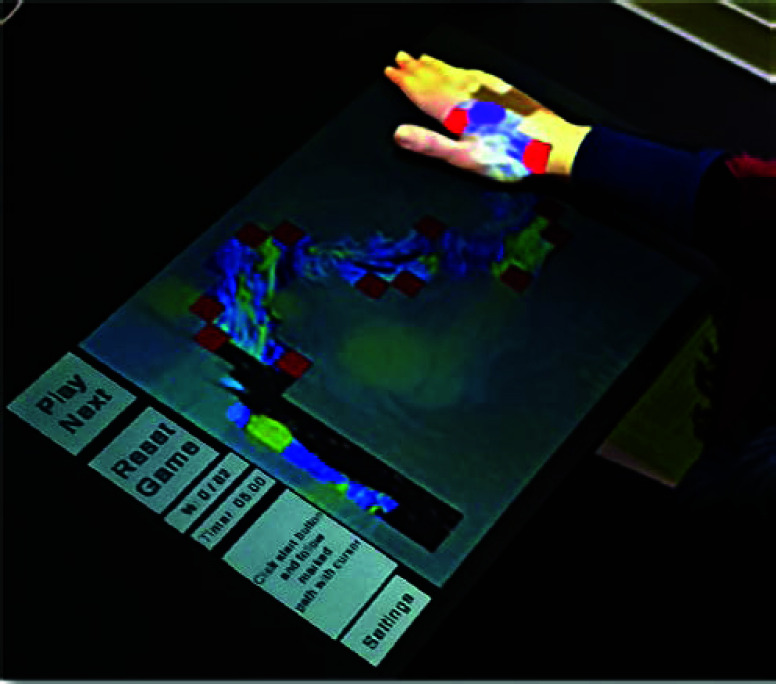
User playing the game on the projected-AR game screen.

“Painting Discovery” SG involves a planar exercise performed with one hand on a surface, utilizing shoulder and hand movements to train flexion extension within the normal range of motion of 0-130°. The patient moves a virtual cursor, controlled by hand movements, along a predefined 2D trajectory projected onto a desk. As the cursor passes over various tiles that make up the trajectory, their transparency changes, revealing parts of a hidden painting, which is fully displayed once the player completes the trajectory (Figure 2). The user executes each trajectory three times, resulting in nine exercises. There are three trajectories representing three levels of difficulty: single arc (easy), double arc (intermediate), and infinity symbol (difficult).

The single and double arc trajectories are based on the “Rolyan arc” range of motion [22], a tool for shoulder rehabilitation [23]. Our game, ‘Painting Discovery,’ is not a standalone solution but a complementary tool in the overall rehabilitation process. It aims to make the rehabilitation process engaging and motivating for patients. The SG incorporates conventional physiotherapy principles for treating upper limb deficits that affect ROM. The game provides visual feedback, including metrics such as speed, acceleration, normalized jerk, and completion time, to help patients track their progress. This progress-tracking feature instills confidence in the technology’s effectiveness. The visual feedback includes specific information such as instructions, completion times, and motivational messages (i.e. “ Fantastic effort! Your range of motion is improving!”) during rehabilitation sessions.

The game is adaptable to different patient needs through software and hardware adjustments. Therapists can modify trajectory path and timing, while friction can be adjusted using different desk pad materials. The game is suitable for all ages, with the option to replace the background painting with a cartoon-style image for children. It also features background music for an immersive experience.

Before starting the game, a configuration panel allows initial adjustments such as cursor calibration, volume, and brightness settings. The low cost and portability of the SG enhance its suitability for game-based therapy, particularly in clinical and home settings.

### B. Participants

Twenty-three subjects between 21 and 65 years of age were involved in the study, divided into two groups: sixteen healthy subjects (8 physiotherapists and 8 engineers) and seven pathological subjects (patients with shoulder pathology). All participants provided informed consent after receiving details about the study. Table 1 summarizes the demographics of the two groups, including their experience with VGs and AR, as well as information on upper limb pathology and if they underwent to surgery.The pathological subjects exhibited varying degrees of functional limitations, measured as a percentage disability on the QuickDASH (see section D in Material and Methods), and different pathologies.

**TABLE 1:** Demographics of Participants

	Healthy Subjs.	Pathological Subjs.
Total Subjects	16	7
Gender (male, female, non-binary)	5, 11, 0	2, 5, 0
Age (min, max, mean, STD)	21, 46, 31, 7	30, 65, 54, 11
Dominance (L, R, ambid)	0, 16, 0	0, 7, 0
Vision (10/10 naked eye, 10/10 corr.)	7, 9	1, 6
VG Exp. (0, limited, familiar, expert)	2, 5, 8, 1	5, 1, 1, 0
AR Exp. (0, limited, familiar, expert)	4, 3, 7, 1	5, 1, 1, 0
Affected Limb (0, left, right, both)	16, 0, 0, 0	0, 1, 5, 1
Shoulder Pathology *	-	1, 3, 1, 1, 1
Surgery	-	0
* (rheumatoid arthritis, bursitis, subacromial impingement, rotator cuff tear, calcific tendinopathy)

We used this metric to identify an ideal population, considering that shoulder rehabilitation addresses issues common across various pathologies. Inclusion criteria for both groups were: age from 18 to 65 years, vision of 10/10 with or without correction, no shoulder pathology or pain for healthy subjects, shoulder pathology about to start at least six weeks of conventional physiotherapy (one session per week with a physiotherapist plus daily home exercises), and no contraindications for performing physiotherapy exercises for shoulder ROM for pathological subjects.

Exclusion criteria for both groups were: central and/or peripheral nervous system pathologies that impair movement, cognition, and/or vision (e.g., neoplastic, vascular, traumatic, compressive, infectious, inflammatory, demyelinating, or neurodegenerative diseases), pathologies or conditions limiting head movements (e.g., cervical vertebral stabilization), pathologies or conditions limiting the functionality of the upper limb under examination, aside from the shoulder pathology in the second group (e.g., post-traumatic elbow arthrosis, wrist arthrodesis).

### C. Experimental Procedure

The study was conducted in two distinct phases, each with its specific purpose. The first phase was designed to establish a baseline by recruiting and testing healthy subjects. In the second phase, we shifted our focus to a group of subjects with shoulder pathologies who were about to start a physiotherapy program at the rehabilitation center. These patients underwent two assessments: the first during the acute phase before initiating conventional physiotherapy (baseline assessment, T0) and the second after six weeks of traditional physiotherapy (post-intervention assessment T1). This timeline is consistent with methodologies employed in similar studies [13], [14].

The physiotherapy treatment included manual therapy, mobility exercises, and muscle strengthening with isotonic exercises. Some exercises were performed under the supervision of a physiotherapist, while others were prescribed for daily independent practice at home. In addition, depending on the patient’s needs, some laser therapy sessions could be scheduled.

The test protocol used in subjects with shoulder pathology was as follows:

**T0** - This procedure lasted 20 min, including:

1. Patient reception, introductions, study overview, signing of informed consent [2 min].
2. Completion of the QuickDASH (see section D in Material and Mehods for questionnaires) [2 min].
3. Completion of the ad hoc questionnaire, including the background information section (demographics; see Table 1) and a pre-test pain assessment using the NRS (Numerical Rating Scale) (see Section D in Material and Mehods). [2 min].
4. SG explanation [3 min].
  a. Virtual cursor operation and possible difficulties.
  b. Objective of the game.
  c. Description of exercises, GUI (Graphical User Interface), and feedback.
5. Patient positioning and cursor calibration [2 min].
  a. Patient sitting on a chair, at proper distance from the table so that the whole frame is in hand range, without touching the backrest with the elbow, and with back always against the backrest, soft arm in front of him with his hand resting on the surface.
  b. Ensure the patient has no rings or bracelets, is comfortable, and can easily move the hand on the surface.
  c. Instructing patient to maintain this position during all exercises, playing using the upper limb while avoiding upper body compensation.
  d. Perform virtual cursor calibration.
6. Performing the exercises [5 min].
  a. Warn the patient about the start of the test.
  b. Perform each task 3 times: single arc, double arc, infinity symbol.
  c. Notify the patient of the end of the test and the possibility of viewing the statistics.
7. Completion of the 14 questions from the ad hoc Likert *Gaming Engagement and Ergonomics* questionnaire (see section D in Material and Methods) and one post-test pain of the upper limb using the NRS, [4 min].

The T1 protocol follows the same structure as T0 but without Step 1. It includes Steps 2, 3, 4, 5, 6, and 7, with Steps 3 and 7 modifications. In Step 3, participants provided only their full names and repeated the NRS pain assessment. In Step 7, they completed five questions from the ad hoc Likert Satisfaction Questionnaire (see Section D) and one question about post-test pain (NRS) 1 min].

We tested the healthy group using the T0 protocol, without administering the upper limb pathology questionnaire, the QuickDASH, or the NRS to this group.

### D. Questionnaires

Ad-hoc questionnaire for the healthy group:

a. *Background Information*: 10 questions on age, vision, experience with VGs and AR, hand dominance, and any pathologies or upper limb pain.
b. *Assessment of Gaming Engagement and Ergonomics*: 14 questions on motivation, engagement, and technical features. Responses were recorded on a 5-point Likert scale: strongly disagree (1) to strongly agree (5).

The questionnaire for the pathological group included:

1. *QuickDASH Questionnaire*: Assessing upper limb disability with 11 questions on daily activities Performed the last week. Responses range from 1 (“no difficulty”) to 5 (“I could not do it”). Results were processed using formula (1) to yield a percentage value of upper limb disability. QuickDASH is a shorter version of DASH (Disability of the Arm, Shoulder, and Hand), including 30 mandatory and 8 optional questions. QuickDASH was chosen for its shorter testing time while maintaining validity [24]:
\begin{equation*} \left ({{ \frac {sum ~of~n ~responses}{n}-1 }}\right )\times 25=score \tag {1}\end{equation*}
  where *n* is the number of completed items.
  We conducted a correlation analysis to explore the relationship between functional disability improvement (QuickDASH scores) and subjective measures, including Engagement, Ergonomics, and Satisfaction. Due to the small sample size (n = 4), we used Kendall’s Tau-b correlation in SPSS. The $\Delta $QuickDASH (pre-post difference) was used to assess improvement in functional disability. Engagement and Ergonomics were measured at T0, Satisfaction was measured at T1.
2. *NRS.* The eleven-point NRS was used to evaluate pain perception in subjects with shoulder pathology, measuring the impact of the SG on pain levels. Response ranges from 0 (no pain) to 10 (extreme pain) [25], [26]. Measurements were taken at T0 (pre- and post-exercise) and T1 (pre and post-exercise) to track changes in pain. Data were analyzed using the Wilcoxon Signed-Rank Test to compare NRS scores across various time points. This non-parametric test was selected due to the small sample size, and a p-value of <0.05 was deemed statistically significant.

*3 Ad hoc Questionnaire including:*

a. *Background Information*: 8 questions on age, vision, and experience with VGs and AR.
b. *Upper Limb Information*: 6 questions on hand dominance, pathology, and upper limb pain, including pain assessment pre- and post-exercise using an NRS.
c. *Assessment of Gaming Engagement and Ergonomics*: 14 questions on motivation, engagement, and technical features, rated on a 5-point Likert scale, from 1 (strongly disagree) to 5 (strongly agree).
d. *Evaluation of Satisfaction*: 5 questions on enjoyment and comparison with conventional physiotherapy, rated on a 5-point Likert scale, similar to other studies [13].

The statistical analysis of ad-hoc questionnaires was conducted using SPSS Statistics Base 22 software. Medians and interquartile ranges were used due to the small sample size and non-normal data distribution. To ensure the robustness of our methodology, we used the Kruskal-Wallis test to evaluate whether response patterns varied based on participants’ health status (healthy or pathological) and their experience with video games and AR, categorized as no experience, limited, familiar, and expert. The test was conducted separately for the healthy group, the pathological group, and the combined sample. A p-value of <0.05 was considered statistically significant. The Wilcoxon test was used to assess whether participants’ overall responses to Engagment and Ergonomics questions deviated significantly from random patterns, without differentiating between groups. A p-value of <0.05 was also used to indicate statistical significance.

## III. Results and Discussion

Of the 7 patients enrolled in the study, all participated in the initial session, but only 4 completed the second session. Three participants discontinued their involvement in the study; 2 withdrew from physiotherapy before completing the 6-week course. They were consequently excluded from further participation, while 1 completed physiotherapy but did not participate in the final evaluation due to personal reasons. However, it was possible to administer the final evaluation questionnaire to 2 of them remotely. Therefore, the data comparing pre- and post-conventional physiotherapy outcomes includes only patients who completed both study phases. We considered the data from the initial trials of the participants with shoulder disorders to compare healthy subjects and those with pathology.

### A. Questionnaires

#### 1) Ad-Hoc Questionnaire

The study presents results on engagement and ergonomics questionnaires, administered to both groups at T0, in Tables 2 (which shows the results of the Wilcoxon test) and III (which presents the Kruskal-Wallis test), and satisfaction findings, administered to the pathological group at T1, in Table 4. We rated a substantial agreement across most items. Notably, the Wilcoxon signed rank test revealed a non-significant result (p = 0.414) for the third item of the engagement questionnaire, ‘Visual feedback such as the timer and scoring system is motivating,’ with a median response of 3 (‘neutral’), indicating no significant difference. This outcome may be due to the subjective nature of the item and the underappreciation and difficulty in interpreting visual feedback. Feedback consists of numerical values for speed, acceleration, completion time, and Normalized Jerk; future developments will enhance this by incorporating performance rankings and visualized goals. We found all the other responses were consistent. In the Kruskal-Wallis test, experience with VGs or AR did not influence responses in either group, suggesting consistent evaluation of the SG “Painting Discovery” regardless of technology familiarity.

**TABLE 2:** Engagement and Ergonomics Likert Questionnaire Results, Wilcoxon Test

ITEM		Median (25°-75°)			Wilcoxon test
		All	Pathological Subjs.	Healthy Subjs.	$p$ - value
Engagement	Game goal (discovering the painting) is motivating, interesting, and engaging.	4 (4-5)	4,5 (4-5)	4 (4-4)	0,000
	Game goal is clear.	5 (4-5)	5 (5-5)	4,5 (3,8-5)	0,000
	Visual feedback such as countdown timer and scoring system is motivating.	4 (3-4)	4 (3-4)	3 (3-4)	0,414
	Game visuals and audio are enjoyable.	4 (4-5)	4,5 (4-5)	4 (4-5)	0,001
	Likely to play again.	4 (4-5)	4 (4-5)	4 (3,8-4)	0,000
Ergonomics	Graphical User Interface is intuitive and user-friendly.	5 (4-5)	5 (4-5)	4,5 (4-5)	0,000
	Text instructions, buttons, and counters are readable and clear.	4 (4-5)	4,5 (4-5)	4 (4-4,3)	0,000
	Adjusting the projected image brightness and volume improves playability.	4 (3,8-5)	4,5 (3,25-5)	4 (3,8-4)	0,014
	Trajectory thickness and panel size allow good playability of the game.	4 (4-5)	4 (4-5)	4 (4-4,3)	0,000
	Projected image is well contrasted to allow for good playability.	4 (4-5)	4 (4-5)	4 (4-4)	0,000
	Projected image has a good resolution to enable good playability.	4 (4-5)	4,5 (4-5)	4 (4-4,3)	0,000
	Latency between real hand movement and virtual 3D cursor displacement is acceptable.	4 (4-5)	4 (4-5)	4 (3-4)	0,004
	Interaction with the game does not require mental effort.	4 (4-5)	4 (4-5)	4 (2,8-4,3)	0,004
	No postural discomfort (arm-shoulder excluded) is perceived during the game session.	4 (3-5)	5 (4-5)	3 (2,8-4,3)	0,022

**TABLE 3:** Engagement and Ergonomics Likert Questionnaire Results, Kruskal-Wallis for VG and AR Parameters

QUESTION		p-value < 0.05					
		All		Pathological Subjs.		Healthy Subjs.	
		VG	AR	VG	AR	VG	AR
Engagement	The game goal (discovering the painting) is motivating, interesting, and engaging	0,243	0,539	0,846	0,846	0,103	0,885
	The game goal is clear	0,734	0,716	0,292	0,292	0,442	0,312
	The visual feedback such as countdown timer and scoring system is motivating.	0,801	0,913	0,466	0,466	0,115	0,268
	The game visuals and audio are enjoyable.	0,074	0,553	0,155	0,155	0,169	0,834
	Likely to play again.	0,252	0,595	0,929	0,929	0,228	0,790
Ergonomics	The Graphical User Interface (buttons) is intuitive and user-friendly.	0,330	0,418	0,417	0,417	0,387	0,634
	The text instructions, buttons, and counters are readable and clear.	0,418	0,398	0,929	0,929	0,226	0,228
	Adjusting the projected image brightness and volume improves playability.	0,461	0,779	0,319	0,319	0,521	0,816
	The trajectory thickness and the panel size allow good playability of the game.	0,353	0,639	0,094	0,094	0,659	0,932
	The projected image is well contrasted to allow for good playability.	0,900	0,353	0,130	0,130	0,350	0,218
	The projected image has a good resolution to enable good playability.	0,126	0,091	0,211	0,211	0,168	0,200
	The latency (lag, delay) between real hand movement and virtual 3D cursor displacement is acceptable.	0,874	0,836	0,364	0,364	0,256	0,932
	Interaction with the game does not require mental effort.	0,232	0,811	0,352	0,352	0,361	0,929
	No postural discomfort (arm-shoulder excluded) is perceived during the game session.	0,194	0,204	0,397	0,397	0,763	0,612

**TABLE 4:** Satisfaction Likert Questionnaire Results

QUESTION	Pathological Subjs. Median (25°-75°)
The game “Painting Discovery” is pleasant.	4 (3,5-4,5)
This system provides a more motivating experience compared to traditional physiotherapy.	3(3-4)
Using this system would motivate me to pursue traditional physiotherapy as well.	3(3-4)
If possible, I would use this system at home with an exercise program.	4 (3-4)
I feel confident using the system independently, even without a supervisor.	4 (3,5-4,5)

Median responses were predominantly positive, affirming strong agreement in both groups, underscoring the SG’s ergonomic and engaging nature, even for subjects with shoulder pathology.

The satisfaction questionnaire (Table 4) among the pathological group confirmed that “Painting Discovery” is enjoyable and desirable for home use in exercise programs, aligning with high engagement results.

While patients expressed confidence in using the system independently, they remained neutral when comparing it with conventional physiotherapy. These findings highlight an interest in pathological subjects in integrating “Painting Discovery” with physiotherapy.

#### 2) Numeric Rating Scale (NRS)

Four participants completed both pre- and post-exercise assessments at all time points. The Wilcoxon Signed-Rank Test was applied to evaluate changes in NRS scores across the time points. In all comparisons, the p-values were greater than 0.05, indicating no statistically significant differences between the time points. According to previous studies [22], [27], a reduction of at least two points on the NRS scale represents the MCID, the smallest change perceived by the patient, indicating a clinically significant improvement in pain. Only two subjects showed an MCID change of at least two points (never exceeding two points) (Table 5). One subject experienced a reduction in pain, while the other reported an increase. These two subjects completed both the initial trial and the test after six weeks of physiotherapy, with a clinically significant difference in NRS observed in only one case. Although both subjects met the MCID threshold, the interpretation of significance also considers clinical context and individual variability.

**TABLE 5:** Numeric Pain Rating Scale (NRS) Scores for Each Subject Before and After Exercise at T0 and T1

Patient ID	T0 ${}_{\mathrm {Pre}}$	T0 ${}_{\mathrm {Post}}$	T0 ${}_{\mathrm {Pr -}}$T0 ${}_{\mathrm {Post}}$	T1 ${}_{\mathrm {Pre}}$	T1 ${}_{\mathrm {Post}}$	T1 ${}_{\mathrm {Pre}}$-T1 ${}_{\mathrm {Post}}$
1	2	1	1	4	6	−2
2	1	2	−1	0	0	0
3	4	5	−1	3	3	0
4	3	1	2	1	1	0

For example, the participant who showed pain reduction may have been positively influenced by their enthusiasm for using SG for the first time.

We are not assessing chronic pain outcomes but the impact of an exercise regimen involving activity levels comparable to or higher than daily activities. Literature [27] suggests that increased activity can heighten pain perception in individuals with shoulder pathology, so the lack of significant differences before and after exercises may indicate a neutral or positive outcome, consistent with favorable results in ergonomics, engagement, and satisfaction. This suggests SG could be a promising tool for managing pain perception in these patients.

The flexion-extension movement on the horizontal plane, performed while resting on a surface, might be relatively easy for some patients. This lack of challenge could mean that for those who did not experience increased pain, the test did not represent a significant increase in activity level.

In any case, the “Painting Discovery” SG does not significantly change pain perception. Since exercise involves a higher level of activity than resting, an increase in pain perception might be expected, which occurs in some cases. However, this is not the norm, and subjective factors may influence the perceived pain, either not increasing or decreasing it. Studying these factors could provide an interesting avenue for verifying whether increasing the activity level (e.g., more difficult tasks, such as those on a sagittal/vertical plane) keeps the pain constant. Additionally, enhancing aspects of SG that could reduce pain perception could make the experience even more enjoyable for patients, potentially revolutionizing the field of physiotherapy and rehabilitation.

#### 3) Quickdash

We evaluated the upper limb disability in pathological subjects before and after rehabilitation treatment to evaluate initial conditions and determine whether the improvement from conventional physiotherapy correlates with the engagement, ergonomics, and satisfaction tests and the performance in the SG (see section B in Results).

The initial disability levels varied ranging from 22.73% to 52%. Three subjects dropped leaving only four for post-treatment evaluation.

According to Franchignoni et al. [28] an MCID for the QuickDASH, of 15.91 is significant. Among the four patients who completed the study, all showed improvement but only two met the MCID threshold as shown in the table6.

**TABLE 6:** Pathological Group Data With QuickDASH Scores (pre, post, and $\Delta $QuickDASH), Engagement, Ergonomics, and Satisfaction Scores

Patient ID	1	2	3	4
QuickDASH T0	43,18	52,27	29,55	47,73
QuickDASH T1	36,36	11,36	27,27	15,91
$\Delta $QuickDASH	6,82	40,91	2,27	31,82
Score Engagement	5	4	4	4
Score Ergonomics	4	4	4	4
Score Satisfaction	4	4	3	4

Notably, these two patients initially had the highest disability (~50%), suggesting that greater initial impairment may allow for more substantial recovery.

Correlation analysis revealed a strong positive association between QuickDASH improvement ( $\Delta $QuickDASH) and Satisfaction (Kendall’s Tau-b correlation $\tau = 0.707$, p = 0.180), indicating that greater functional recovery tends to align with higher satisfaction, though the result was not statistically significant due to the small sample size. In contrast, engagement and ergonomics did not show strong relationships with recovery.

Overall, these findings highlight that clinically significant improvement is possible within six weeks of physiotherapy, particularly in patients with greater initial impairment. This underscores the importance of targeted rehabilitation for severe cases and suggests that perceived progress, rather than absolute post-treatment function, plays a key role in patient satisfaction.

### B. Motor Performance Measured through the SG

The “Painting Discovery” system offers a comprehensive measure of motor performance, including completion time, speed, acceleration, and normalized jerk, which measures movement fluidity. This comprehensive approach is promising for applications in motor performance assessment.

#### 1) Comparison Before and After Conventional Physiotherapy

One of the interests of the study is to assess potential improvements in the pathological limb after traditional physiotherapy. Data comparing T0 and T1 are limited, as only four patients completed the study, with two showing significant improvement in upper limb disability.

The average completion time decreased from $67.28~\pm ~41.85$ seconds at T0 to $53.83~\pm ~29.57$ seconds at T1, while speed increased from $0.052~\pm ~0.021$ m/s to $0.056~\pm ~0.017$ m/s. Acceleration improved from $0.91~\pm ~0.45$ m/s^2^ at T0 to $1.00~\pm ~0.81$ m/s^2^ at T1, and Njerk showed a significant reduction from ( $5.62~\pm ~10.58$) $\times 107$ m/s^3^ to ( $1.57~\pm ~3.71$) $\times 107$ m/s^3^, indicating smoother and more controlled movements at T1.

A Wilcoxon Signed-Rank Test was conducted to analyze these differences further to compare key performance metrics. Significant improvements were observed in completion time (p = 0.025) and normalized jerk (p = 0.018), indicating enhanced movement efficiency and control. No significant changes were found in speed or acceleration (p > 0.05), suggesting that patients maintained consistent movement intensity while becoming more efficient. These improvements in SG performance did not correlate significantly with changes in the QuickDASH score, as determined by Spearman’s Rho correlation analysis. This discrepancy may be due to the different ways each tool measures function—QuickDASH assesses overall disability in daily activities, while the SG system measures motor performance during specific controlled tasks.

An important consideration in this study is the technological advancement of the SG system, which now includes a planar task on the transverse plane involving horizontal flexion-extension movements. While this task can provide valuable insights into motor control for some shoulder conditions, it may be too challenging or not fully representative of daily functional challenges for patients with severe restrictions, such as those with adhesive capsulitis or post-surgical conditions. This could limit the relevance of the SG performance metrics for this group of patients.

Future updates are already under consideration to make these differences more discernible:

• Move the SG play panel to a vertical plane, combining horizontal flexion-extension with sagittal plane movements, increasing task difficulty. This makes the motor gesture required to play representing a moderate-to-intense activity level suitable for a wide range of shoulder pathologies. Moreover, positioning the exercise on a vertical plane allows for adjusting the movement’s height, potentially stressing the ROM up to the maximum degrees of flexion if necessary.
• Evaluate the integration of new performance parameters, like the Log Dimensionless Jerk (VDLJ), is one of the latest proposed measures in the Jerk family of parameters that is dimensionless (does not depend on measures of movement amplitude and duration) and solves problems related to variability [29].

#### 2) Comparison Between Healthy and Pathological Subjects

Even if the sample is small we decided to evaluate the mean and standard deviation for both groups at T0, as another aim of this study is to determine if the system can differentiate between healthy and pathological subjects using the current performance parameters(Completion Time, Speed, Acceleration, NJerk).

Table 7 evidences that the pathological group had significantly longer completion times compared to the healthy group. There was no significant difference in speed between the groups. However, the pathological group exhibited higher acceleration and jerk, indicating impaired motor control.

**TABLE 7:** Performance Parameters in Healthy and Pathological Subjects

Performance Parameters	Healthy Subjs. (mean± sd)	Pathological Subjs (mean± sd)
Completion Time (s)	39.02s±24.21	56.49s±37.85
Speed (m/s).	0.056	0.059
Acceleration (m/s^2^)	$0.78~\pm 0.56$	$1.12~\pm ~0.93$
NJerk (m/s^3^)	$6.68\times 107\pm ~1.37\times 108$	$9.22\times 106\pm 2.51\times 107$

We tested the normality of the distribution using the Shapiro-Wilk test before conducting the Mann-Whitney U test to compare performance metrics between the groups at T0. The results showed that the pathological group had significantly longer completion times ( $p < 0.001$), lower acceleration ( $p < 0.001$), and higher jerk ( $p = 0.025$), confirming impaired motor control compared to the healthy group. However, there was no significant difference in speed between the two groups at T0.

## IV. Conclusion

This study assessed the feasibility and effectiveness of *Painting Discovery*: a projected AR-based SG for shoulder rehabilitation. The system was confirmed to be engaging and ergonomic for both healthy and pathological subjects regardless of prior experience with VGs or AR, with strong patient interest in integrating it into home rehabilitation programs.

Motor performance analysis revealed significant differences between healthy and pathological subjects, particularly in completion time, acceleration, and jerk. Clinical improvements were noted in completion time and normalized jerk following physiotherapy, indicating enhanced movement efficiency. However, speed and acceleration remained unchanged, and these performance improvements did not significantly correlate with QuickDASH scores.

Despite promising results, the study’s small sample size limits broader conclusions. Additionally, the system currently focuses on horizontal flexion-extension movements, which may not fully address all shoulder rehabilitation needs. Future developments should incorporate vertical-plane movements and additional performance parameters to enhance clinical applicability.

Overall, *Painting Discovery* demonstrates strong potential as a motivating and accessible rehabilitative aid, as well as an instrument to evaluate patient progresses thorough the rehabilitation journey. Further research with a larger sample size and expanded movement assessment is necessary to refine its clinical relevance and effectiveness.

## Author Contributions

Conceptualization, Rosanna Maria Viglialoro, Marina Carbone, Giuseppe Turini, and Paolo Parchi; Data Curation, Rosanna Maria Viglialoro, Marina Carbone, Donato Gallone; Investigation, Vincenzo Ferrari, Marco Gesi, and Paolo Parchi; Methodology, Rosanna Maria Viglialoro and Marina Carbone, Giuseppe Turini, Sara Condino, Vincenzo Ferrari and Paolo Parchi; Supervision, Rosanna Maria Viglialoro and Marina Carbone; Validation, Rosanna Maria Viglialoroand and Marina Carbone; Writing—Original Draft, Rosanna Maria Viglialoro; Writing—Review and Editing, Rosanna Maria Viglialoro, Marina Carbone, Giuseppe Turini, and Sara Condino. All authors have read and agreed to the published version of the manuscript.
